# Systems Modelling of NHEJ Reveals the Importance of Redox Regulation of Ku70/80 in the Dynamics of DNA Damage Foci

**DOI:** 10.1371/journal.pone.0055190

**Published:** 2013-02-06

**Authors:** David Dolan, Glyn Nelson, Anze Zupanic, Graham Smith, Daryl Shanley

**Affiliations:** Institute for Ageing and Health, Newcastle University, Campus for Ageing and Vitality, Newcastle upon Tyne, United Kingdom; Vanderbilt University Medical Center, United States of America

## Abstract

The presence of DNA double-stranded breaks in a mammalian cell typically activates the Non-Homologous End Joining (NHEJ) pathway to repair the damage and signal to downstream systems that govern cellular decisions such as apoptosis or senescence. The signalling system also stimulates effects such as the generation of reactive oxygen species (ROS) which in turn feed back into the damage response. Although the overall process of NHEJ is well documented, we know little of the dynamics and how the system operates as a whole. We have developed a computational model which includes DNA Protein Kinase (DNA-PK) dependent NHEJ (D-NHEJ) and back-up NHEJ mechanisms (B-NHEJ) and use it to explain the dynamic response to damage induced by different levels of gamma irradiation in human fibroblasts. Our work suggests that the observed shift from fast to slow repair of DNA damage foci at higher levels of damage cannot be explained solely by inherent stochasticity in the NHEJ system. Instead, our model highlights the importance of Ku oxidation which leads to increased Ku dissociation rates from DNA damage foci and shifts repair in favour of the less efficient B-NHEJ system.

## Introduction

**Figure 1:**
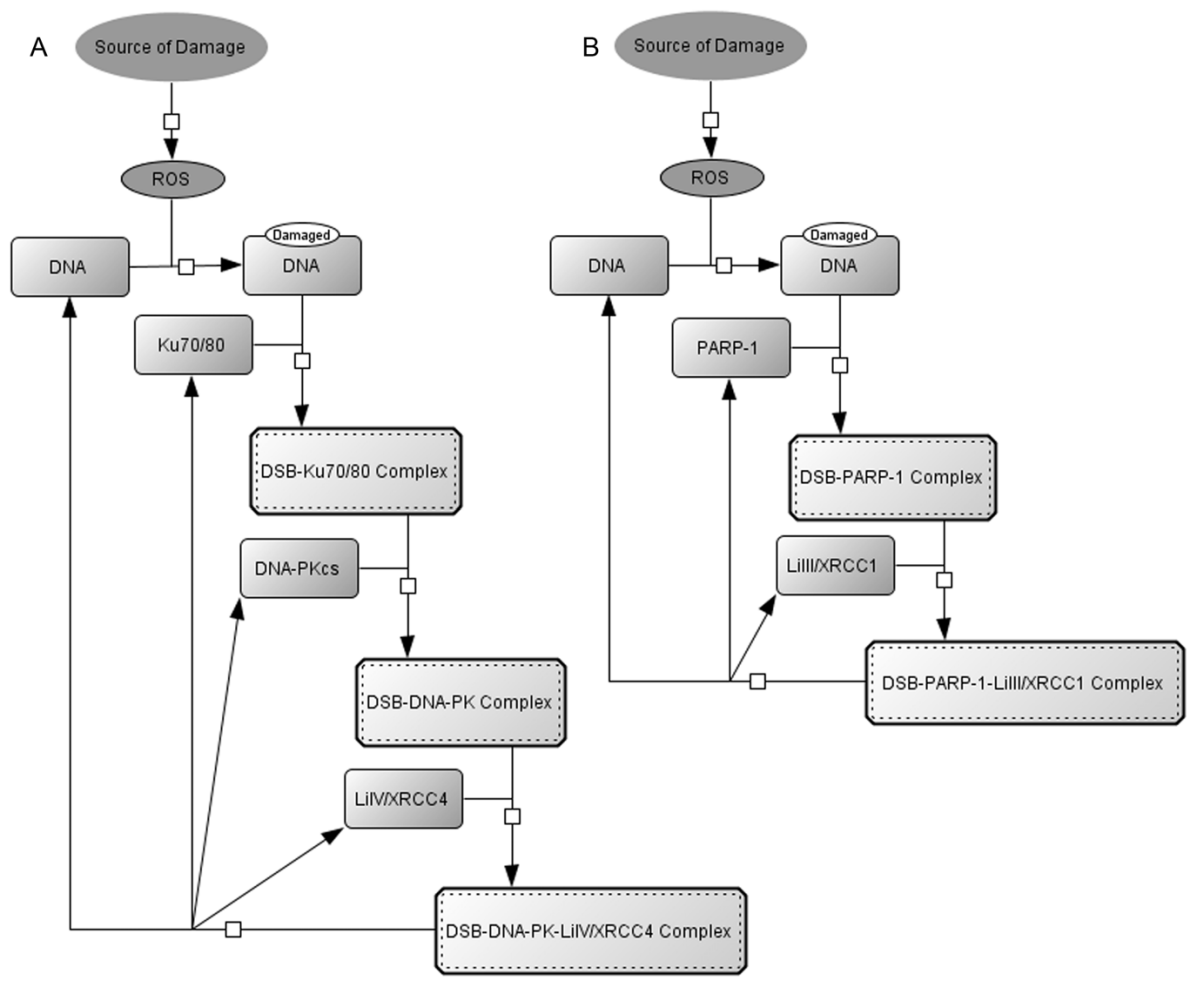
Repair Mechanisms of Non-Homologous End Joining. (A) The primary repair pathway of DSB repair by NHEJ is mediated by a hetrodimer DNA-PK which is made up of Ku70, Ku 80 and DNA-PKcs and is commonly named DNA-PK Dependant Non-Homologous End Joining (D-NHEJ). Once the DNA-PK has formed a complex with the site of the DSB the break is readied for repair by ligation from the Enzyme LiIV which is in complex with XRCC4. (B) A second NHEJ pathway called Backup Non-Homologous End Joining (B-NHEJ) mediated by PARP-1 also exists. Once the break is primed by the formation of the DSB-PARP complex, the broken ends are ligated by the LiIII/XRCC1 complex.

DNA Double-strand breaks (DSB), arguably the most dangerous kind of DNA damage, are caused by reactive oxygen species (ROS) which are produced as a by-product of cellular respiration as well as various environmental stresses. DSBs are repaired by either Homologous Recombination (HR) or Non-Homologous End Joining (NHEJ). HR, the more accurate of the two processes, is used when a sister chromatid is present to act as a template for rebuilding the damaged DNA, whereas NHEJ is used when this is not the case, as for example in the G1 phase of the cell cycle [1]. In mammalian cells NHEJ is thought to be the more important of the two mechanisms [2] given the slower cell cycle compared to other eukaryotes such as yeast. NHEJ uses two competing pathways: the faster and more accurate repair pathway, DNA-PK Dependent NHEJ (D-NHEJ), mediated by Ku, DNA-PKcs and Ligase IV [3] (Figure 1A); and the recently identified slower, more inaccurate Backup NHEJ system (B-NHEJ) [4], [5] mediated by PARP-1 and Ligase III (Figure 1B), which are better known as key components of single strand DNA break repair [6].

Correct handling of DNA damage is essential for a cell’s survival. Cell lines have previously been observed to inaccurately repair 20% to 25% of their DSBs depending on whether the breaks are simple or complex [7]. This faulty repair, potentially as a result of the error prone nature of B-NHEJ [4], [7], [8], can lead to genome instability, which in turn can lead to cell death or the onset of cancer [9] either directly in the affected cell or in its progeny [10]. However, the role that NHEJ plays in the promotion or avoidance of genome instability is not yet entirely understood, and it is possible that factors traditionally linked to accurate repair, such as Ku, may also be linked to mis-joining of breaks [10].

Whilst ROS can produce DSBs, the DNA damage response (DDR) can result in the production of more ROS inside a cell [11]. Moreover, although clearly a cause of damage to DNA (and indeed all other biomolecules), it is becoming increasingly apparent that ROS plays a much bigger role in cell biology as a number of important cellular signalling pathways are redox regulated [12], [13]. Therefore, the levels of ROS inside a cell can have important effects on its activity. A number of key signalling proteins such as PKA, PTP1B and MEKK1 have been identified as being redox regulated through the oxidation of cysteine residues [14]. Interestingly, the heterodimer Ku70/80 displays a dramatic increase in dissociation rate from DNA when in an oxidising environment [15] and it was hypothesised that oxidation of the Cys-493 residue in Ku80 was the potential cause of this. However, it was subsequently found that this residue played at best only a minor role in the redox related binding and dissociation dynamics of Ku [16], although the other cysteines were not tested and the method by which Ku’s binding activity is modified in an oxidising environment is still unclear.

**Figure 2:**
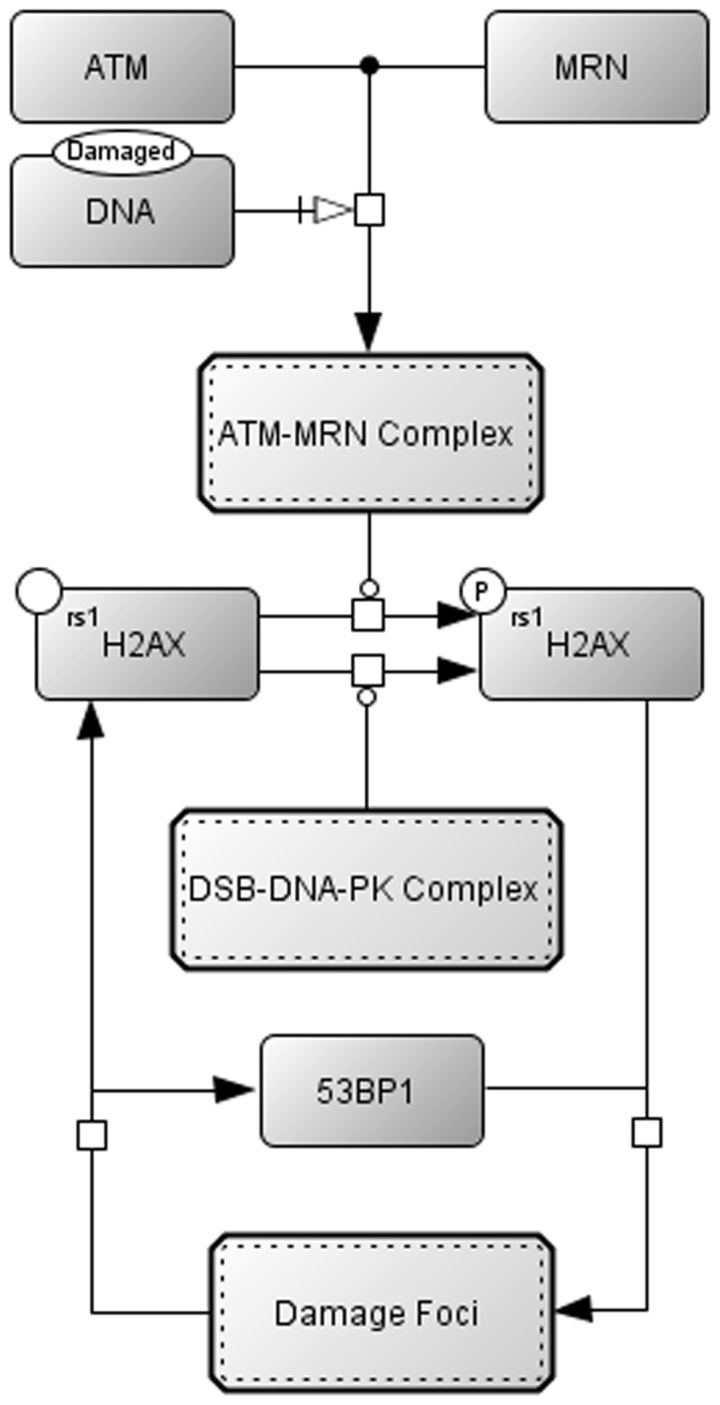
Signalling of DNA double strand breaks is done by the phosphorylation of the histone H2AX and the formation of a Damage Focus around the DSB. Phosphorylation of H2AX is caused by autophosphorylation of ATM and DNA-PKcs at the site of damage.

Whilst much is known about the individual components and the connections that make up NHEJ [1], we know much less of how these components function together dynamically. This understanding can be achieved by dynamic computational modelling using the growing body of experimental data that have become available from time course experiments and other sources [1]. Recently, it has been shown that a cell stressed by gamma irradiation greatly increases its production of ROS [11]. This leads to more DNA damage foci being formed (Figure 2) and a shift in the early repair dynamics, with the number of short lived breaks decreasing significantly after irradiation, as revealed by changes in the “longevity” of recognizable DNA damage foci [17], [18].

The cause of this shift in repair dynamics is currently unclear. Since cellular systems are prone to stochastic effects [19], [20], we hypothesized that the stress-induced shift in distribution of focus longevity is caused by the stochastic nature inherent in the system. In this study, we use a combination of experimental and computational approaches to investigate the cause of the early repair shift. We show that the shift cannot be explained by a model of NHEJ alone, but can by a stochastic model of NHEJ with a redox sensitive D-NHEJ pathway. In addition, we use continuum electrostatics calculations to investigate which of the Cysteine residues in Ku 70/80 may be responsible for its redox regulation.

## Results and Discussion

**Figure 3:**
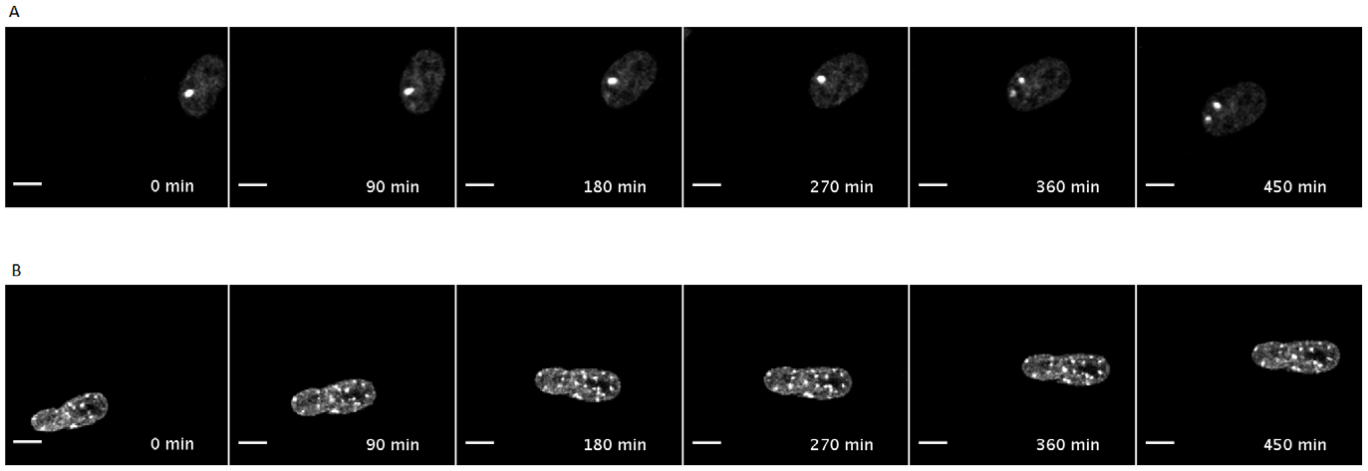
53BP1 Damage Foci induction in human MRC5 fibroblasts. Images of unstressed (A) and stressed (B) cells expressing the fusion protein AcGFP-53BP1c. Scale bar represent 10 µm. See Video S1 and Video S2 for examples foci formation and resolution over time in unstressed and stressed MRC5 fibroblasts respectively.

**Figure 4:**
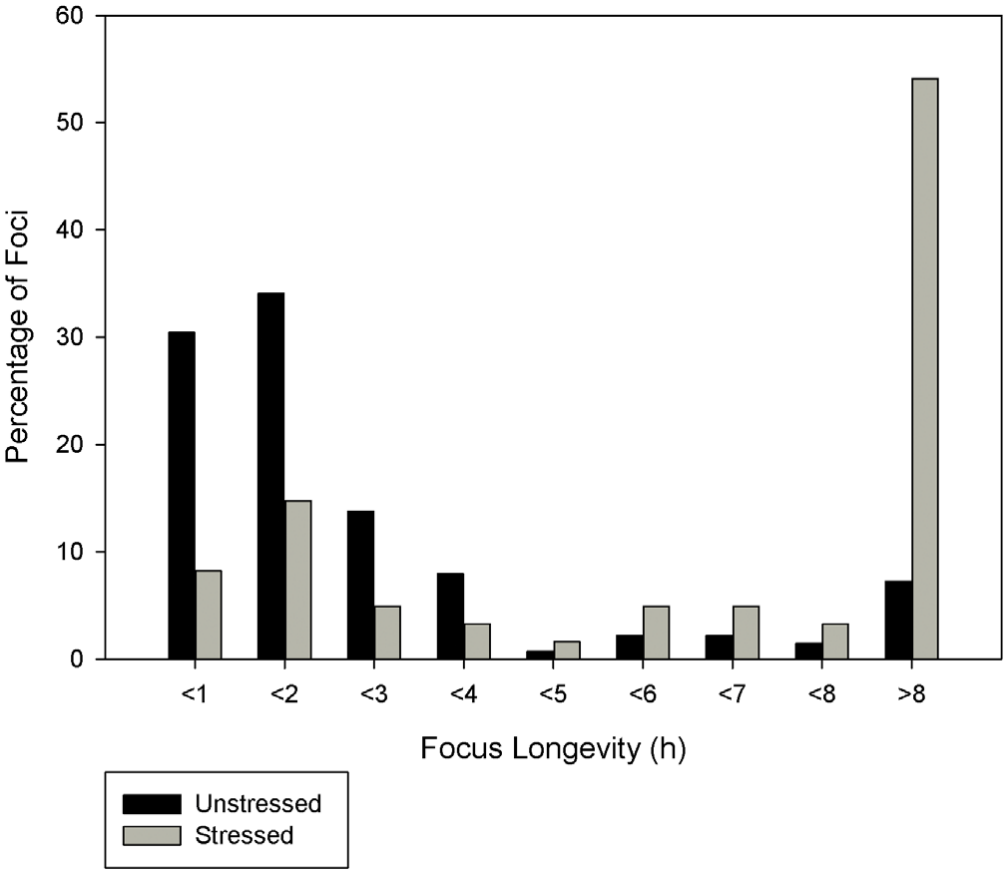
Foci Longevity of live MRC5 cells observed for 30 hours.

When a cell is in an unstressed state, damage foci still form indicating that a cell undergoes some damage when at rest in its typical environment (Figure 3). This is largely because whilst at rest the cell is still subject to mild stresses from its environment and ROS produced by the electron transport chain during respiration. Unstressed MRC5 cells showed a focus emergence rate of 0.53 foci per hour. Over 60% of the foci were repaired in two hours or less (Figure 4) and only 7% survived more than 8 hours of which only a few (3 out of 10) were resolved.

48 hours after treatment with 20 Gy of gamma irradiation the focus rate emergence more than doubled to 1.28 foci per hour and there was a dramatic shift in repair times with 20% of the foci resolved in less than 2 hours and 55% surviving beyond 8 hours (Figure 4) of which only 15% resolved (5 out of 33). Although the number of foci with a lifetime less than 8 hours was greatly reduced in stressed cells, the mode of the distribution in these short lived foci remains the same, favouring repair within 2 hours of the foci forming. Previous work within our labs has shown that cells treated with lower levels of gamma irradiation result in similar damage foci repair dynamics as those treated with 20 Gy but with lower rates of damage foci induction [21], [22].

**Figure 5:**
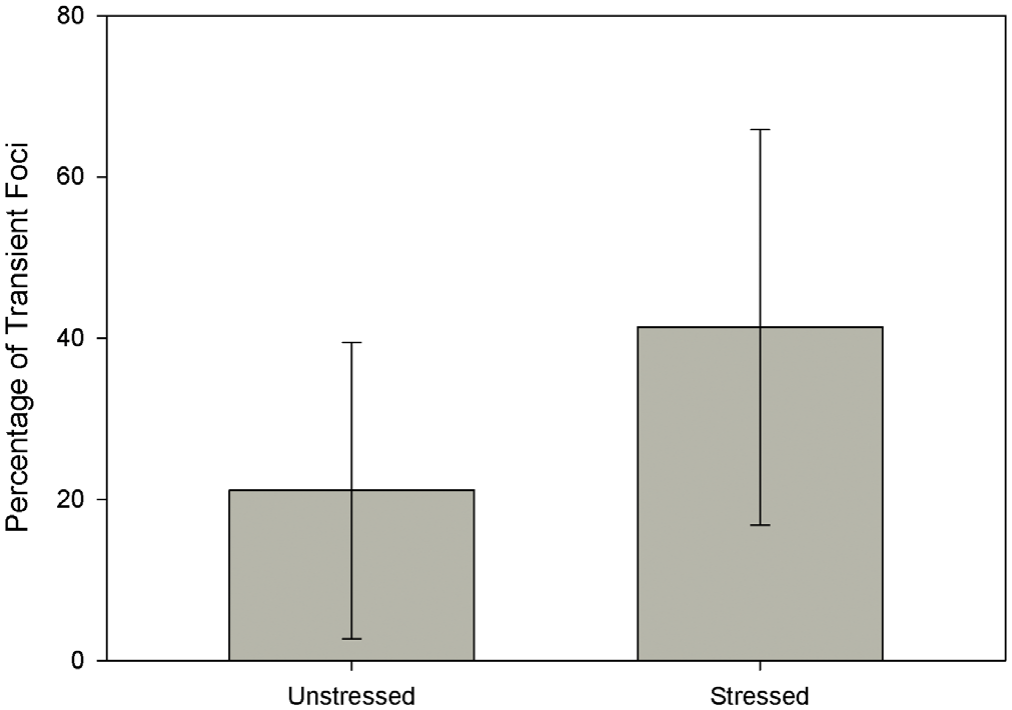
Percentage of transient Foci. These are foci that disappeared and then reformed rather than shrunk to a small size and then regrew. Ten unstressed cells (53 Foci) and 6 stressed cells (135 Foci) were observed in total. Results are presented as mean ± SD.

Since very few damage foci fully resolve once they have lasted more than 8 hours we view them as permanent damage foci. However if our understanding of the NHEJ system as a whole is correct all foci should eventually be resolved. The fact that they are not suggests that either these DSB are irreparable telomeric breaks [23] or there is a downstream effect that feeds back into the NHEJ causing permanence. Transient foci were observed in both resting and stressed live cells although stressed cells had a higher fraction of transient foci on average (Figure 5).

**Figure 6:**
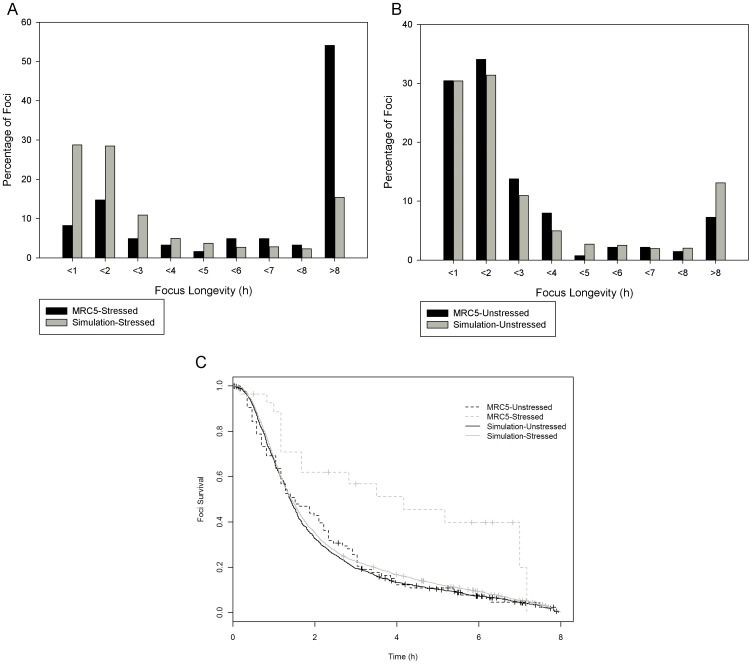
Damage foci longevities in live cells and simulations. (A) Longevities of foci recorded in unstressed MRC5 cells and the unstressed D-NHEJ and B-NHEJ model simulations. (B)Longevities of foci recorded in unstressed MRC5 cells and the stressed D-NHEJ and B-NHEJ model simulations with ROS production increased 2.5 times. Simulated data shows no change other than an increase in the number of breaks produced. (C) Survival curves of short lived foci (8 hours and less) for resting and stressed MRC5 cells (dotted lines) and resting and stressed simulated data (solid lines).

Using the parameters calculated from work within our labs and the data available in published literature the model of the Ku mediated D-NHEJ pathway and the PARP-1 mediated B-NHEJ pathway was found at rest to produce very similar results to the live MRC5 cells with over half the breaks being resolved in less than 2 hours (Figure 6A) and the majority of remaining foci being resolved within 8 hours. Our model not only matched the short term foci dynamics, but also the long term dynamics (those of foci lasting longer than 8 hours) (Figure 6C). Cox regression comparison of simulated and experimental short lived foci survival curves yielded a p-value of 0.65, indicating no significant difference between the model and experiment. Since the focus longevity data was not used in the calculation of the kinetic rates of the model, the matching of the live cell data to the simulation is a positive validation of the unstressed model.

However, increasing ROS production of the unstressed model to represent the stressed state of a live cell 48 hours after being treated with gamma radiation yielded different short term (less than 8 hours) focus longevity distributions than those experimentally observed in the stressed cells and instead appeared to have the same dynamics as the unstressed model (Figure 6B and 6C). From this we can conclude that the change in foci dynamics in stressed cells is not brought about by an increase in the amount of damage alone.

**Figure 7:**
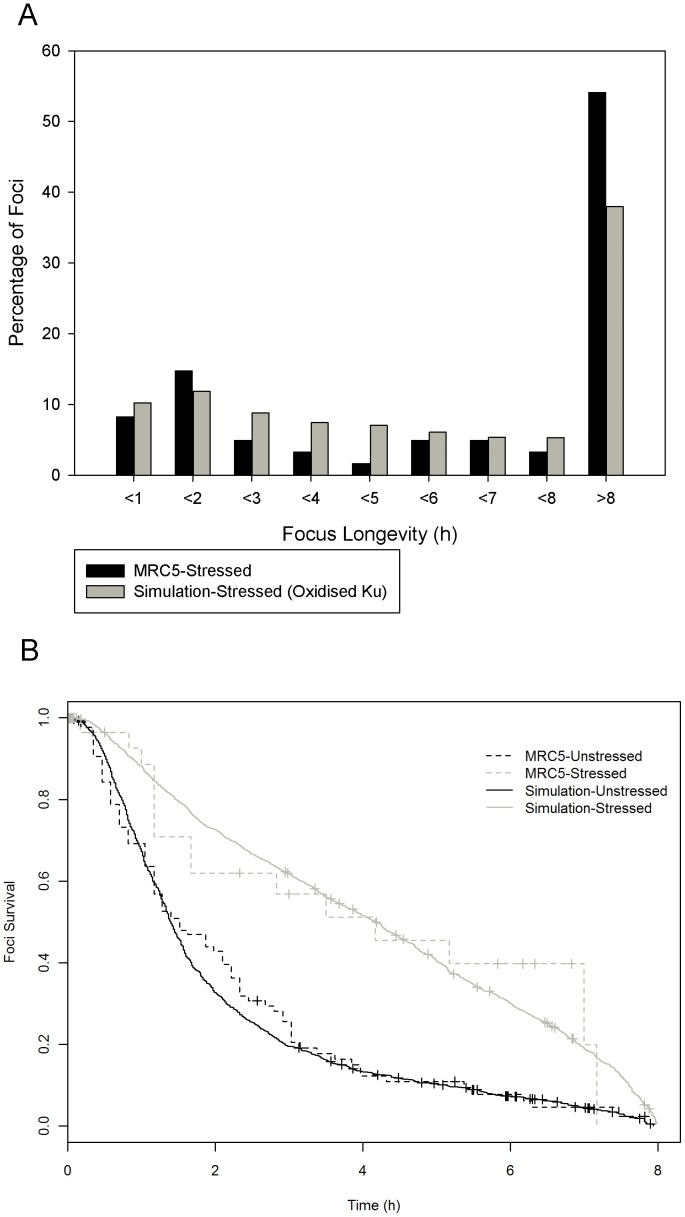
Effects of Ku70/80 redox on NHEJ. (A) Increasing Ku70/80′s and DNA-PK’s dissociation from DNA in line with observations from the literature (15) results in a decrease in short lived foci similar to that of stressed live cell. (B)Survival curves of short lived Foci (8 hours and less) for resting and stressed MRC5 cells (dotted lines) and resting and stressed simulated data (solid lines). Stressed data was collected from the model with increased Ku70/80 dissociation from DNA DSBs.

To what, then, could it be attributed? The Ku heterodimer had previously been shown to have a major shift in dissociation rate from DNA when oxidised [15]. To test whether Ku oxidation had an effect on the dynamics of the model we increased its rate of dissociation from the DSB tenfold in the stressed version of the model [15]. The number of breaks repaired in less than two hours dropped significantly and the number of breaks taking more than 8 hours to repair rose to become similar to stressed live cells (Figure 7A). Cox regression analysis produced a p-value of 0.88 indicating that there is no significant difference in the resolution times of short-lived foci (Figure 7B). This indicates that Ku’s increased dissociation from a DSB, altering repair dynamics due to its redox sensitivity, is enough to explain the observed shift in short term foci dynamics when cells are stressed with gamma radiation.

**Figure 8:**
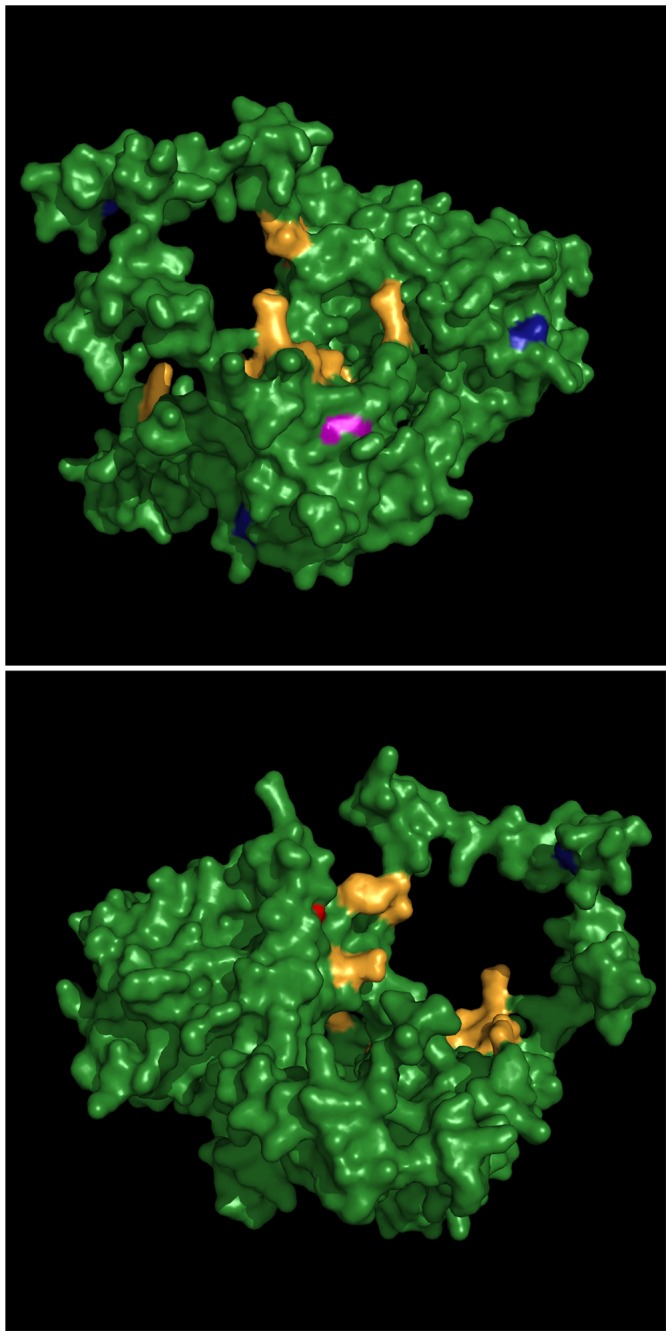
Crystal structure of Ku 80. Images of the front (top) and back (bottom) of the Ku 80 protein displaying the DNA binding domain (yellow), surface cysteines (blue), Cys-493 (red) and Cys-249 (pink).

It was initially thought that Ku’s redox sensitivity and shift in dissociation was a result of the Cys-493 being oxidised; however after mutagenesis experimentation it was concluded that Cys-493 only had a small effect on Ku binding activity [16]. Because the irradiation of cells causes production of large amounts of ROS it is highly plausible that Ku becomes oxidised at the same time that a cell’s DNA is damaged during the treatment. The Cysteine amino acid has a pK_a_ of 8.7 when isolated in solution [24] and a shift in pK_a_ to a value less that 7 suggests that a cysteine residue in a protein is ionisable and therefore a viable target for oxidation [25]. The calculated pK_a_ shifts for Cys-493 in Ku 80 when bound to DNA and unbound show a pK_a_ shift from 8.7 to 9.06 and 7.97 respectively (Table 1). As neither is below 7 our calculations support the findings of [16] in that Cys-493 does not play a significant part in oxidation of the Ku heterodimer. The only surface cysteine to show a large enough drop in pK_a_ to be ionisable is Cys-249 (Figure 8) for which the calculated pK_a_ values are 5.59 and 4.39 when unbound and bound to DNA respectively. Moreover, it is close to the DNA binding site. This, together with the lowered pK_a_ values suggest that the residue could be oxidised with a concomitant effect on DNA binding and is therefore the potential cause of Ku’s observed increase in dissociation from DNA when placed in a oxidising environment [15].

**Table 1:** Pka shift calculation results for the Cysteine residues on the surface of the DNA-PK component Ku 80.

CYS	ΔxferG_A_	ΔxferG_HA_	pka shift
157	−1.49	−5.92	10.63
235	5.54	−0.57	11.36
249	−12.88	−5.73	5.59
296	3.71	−5.28	12.61
346	−0.41	−4.25	10.37
493	−4.89	−5.71	9.06

Cys 157,235, 249 296, 346 and 493 pKa shifts were calculated using the Ku 80 protein binding domain model 1JEQ from the RCSB Protein Data Bank.

Overall our results suggest that the cause of the shift in short term focus dynamics seen in stressed cells is not due to natural stochastic behaviour within a biological system but rather due to an increased rate of dissociation of the heterodimer Ku70/80 from a DSB caused by the oxidising environment within the stressed cell. This increased dissociation alters the competition between Ku and PARP for binding to the DNA, causing the latter to take place more often than it does in an unstressed cell.

Although the apparent competition between D-NHEJ and B-NHEJ can explain the short term NHEJ dynamics it does not explain those of the foci that last longer than 8 hours. We can speculate that the cause of the maintained long lived foci is the result of downstream pro-survival and pro-apoptotic pathways triggered by the presence of the DSB through signalling pathways, such as the p53/p21 signalling that feeds back into the damage repair mechanism further altering how it responds to damage over longer periods of time. When damage is caused, ATM phosphorylates H2AX, which then also influences the p53, p21 and Chk1 pathways which go on to stall the cell cycle and/or trigger apoptosis. At the same time, whilst Ku70 is being used to repair double stranded breaks it is no longer suppressing Bax and its apoptotic function [26], [27], and is no longer inhibiting FOXO4’s cell cycle arrest pathway [28]. In the future we intend to expand our model to take into account these downstream responses and their feedback; and as our model is already The proposed arrays and sets package of SBML level 3 (www.sbml.org), or similar features of rule-based modelling or kappa calculus [29], could be viable ways of carrying this extension out.

Throughout this investigation we have treated D-NHEJ and B-NHEJ as competing systems due to the observed competition between DNA-PK and PARP for binding to a DNA end [30], [31]. However Mitchell *et al.* (2009) hypothesised that PARP and Ku work co-operatively to repair DSBs with 5′ overhangs. The obvious way in which this system would function is that PARP is utilised to loosen the chromatin around the damage site to allow the repair proteins greater access to the site of damage to allow repair to take place. Recent work has also produced evidence of DNA-PK and PARP forming a complex [32] that can bind to the site of damage at the same time. Either way, preliminary modelling of the co-operation of Ku and PARP (results not shown) does not significantly alter the observed dynamics of damage repair proposed in our model. We believe this is because ultimately the ligation of the DSB can only be undertaken by a single ligase enzyme, be it LiIII or LiIV. Given that PARP has roles beyond repair of a DSB and is a potential target in cancer therapy [33], knowing precisely how it functions in the DNA damage response, and how this interaction is regulated, will be of great importance for development of better therapies and is vital to our understanding of how the various systems of DNA repair have evolved.

What is apparent from our work is that DNA repair and, by extension, cell survival is not a straightforward process: rather than a single factor determining the outcome of the damage response, it is more likely the interplay between various mechanisms and processes influences the cell’s response and therefore its survival. This capacity for interplay is clear when the system’s major players and their roles are viewed as a whole.

Although individual components of the entire NHEJ DDR and its downstream effects are quite well understood, how these systems function as a whole is not. What is obvious is that the classical approach to investigating these systems in isolation is not enough; the systems biology approach and creation of large computational models using experimentally derived data delivers a capacity to monitor large scale interactions between known systems that traditional experimentation alone cannot. Our model is the first stochastic model of NHEJ that attempts to model both the D-NHEJ and B-NHEJ pathways as well as the formation of the damage foci and is the first step in producing a large scale systems model of a cell’s response to DNA damage. It has allowed us to rule out that the observed change in foci dynamics could occur without a relative shift in the contributions of the two NHEJ pathways, whilst showing that the redox sensitive change in Ku–DNA binding affecting D-NHEJ provides a plausible mechanism for it.

## Materials and Methods

### 53BP1 Tagging and Live Cell Observation

DSB formation and resolution within a cell was followed by tagging one of the proteins that make up the damage focus created around the site of damage. A plasmid encoding the fusion protein AcGFP-53BP1c was built and expressed in human diploid fibroblast cell line, MRC5, as described previously [21]. For live cell time-lapse microscopy, MRC5 cells were plated in Iwaki glass bottomed dishes (Iwaki), either without treatment (unstressed cells) or after exposure to 20 Gy gamma irradiation (stressed cells). Cells were imaged on an inverted Zeiss LSM510 microscope equipped with a Solent incubator (Solent Scientific) at 37°C with humidified 5% CO_2_, using a 40×1.3 NA oil objective (details in [34], with Z stacks obtained every 10 or 12 minutes for each field as described previously (Passos *et al.*, 2010) for 30 hours. Imaging of stressed cells began 48 hours after treatment. Cells and AcGFP–53BP1c foci were tracked manually using ImageJ (http://rsb.info.nih.gov/ij/); when a focus was formed, the time was recorded and it was tracked through the time course images until it resolved. Some foci were seen to apparently resolve and then reappear at the same position shortly after they disappeared. This dynamic growth and disappearance is a result of the foci being extended by phosphorylation of adjacent H2AX histones and recruitment of flagging proteins such as 53BP1, being dephosphorylated and then dismantled by the Protein Phosphatase2A (PP2A), and then reforming because of the continued presence of the DSB to maintain the signalling of the damage to the rest of the cell. If a focus returned within 2 time frames (24 minutes or less) it was considered a single transient focus rather than two individual foci.

### *In Silico* Modelling

We first constructed a network of the known reactions of D-NHEJ, B-NHEJ and the formation and flagging of Damage Foci using CellDesigner [35]. SBML Squeezer [36] was then used to generate differential rate equations for each reaction using mass action kinetics. Simplified versions of these networks are shown in Figure 1.

When a DSB occurs, typically the heterodimer Ku70/80 (Ku) binds to the broken ends of the DNA followed by recruitment of the DNA-dependent protein kinase catalytic subunit (DNA-PKcs), which together form the complex called the DNA-dependent protein kinase (DNA-PK) [37]. The Ku 70/80 heterodimer is made up from a 70 kDa subunit, Ku70, and an 83 kDa subunit, Ku80. The DNA-PKcs is a large 469 kDa kinase from the family of kinases known as the phosphoinsitide 3 kinase-related protein kinase (PIKK) family [38]. Ku70/80 has a toroid structure which fits over the DNA chain [39] and is thought to provide a platform that enhances the binding of DNA-PKcs to the damaged DNA [40]. It has been shown that Ku70/80 is not always required for the binding of DNA-PKcs [41] but we did not consider this in our model. Following binding, Ku70/80 can either dissociate once more, or form the DNA-PK complex by recruiting DNA-PKcs [16]. The DNA-PK complex then makes a synaptic complex between the two broken ends of DNA to prepare the DNA for re-joining [42] and undergoes autophosphorylation. The break itself is fixed by ligation of the two broken ends carried out by a complex made up of DNA ligase IV and XRCC4 [43], after which all components dissociate. The following equations describe the reactions corresponding to the network connections shown in Figure 1A, see Table S1 for a full list of reactions together with rate parameters.
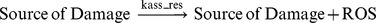


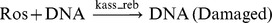


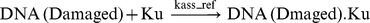


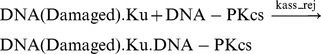


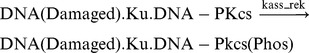


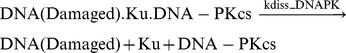





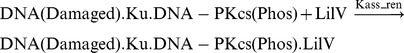


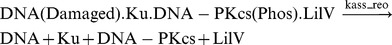


Instead of the DNA-PK complex binding to the site of damage the enzyme Poly [ADP-ribose] polymerase 1 (PARP-1) can form a complex with the double strand break [4], [44] after which Ligase III and XRCC1 are recruited to ligate the break. (Figure 1B, see equations below).



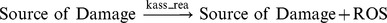





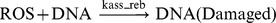





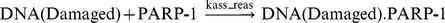





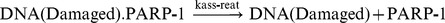





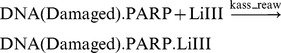





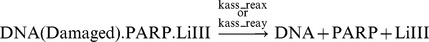



As the repair proteins are being recruited to fix the double stranded break, the Signalling/Flagging system is activated to signal the presence of the damage to a variety of cellular pathways (Figure 2, see equations below). This signalling involves formation of a Damage Focus made up of a number of proteins [45]. It is thought that MRN, a complex of three proteins, Mre11, Rad50 and Nbs1 localises to the site of DNA damage first followed by the phosphoinsitide 3 kinase-related protein kinase ATM (ataxia-telangiectasia mutated) [46]. Previous work has implicated the apoptotic regulator protein Aven as a crucial factor in the activation of ATM at the site of DNA damage [47] which then autophosphorylates [48] and phosphorylates H2AX histones around the DNA damage site [49] (the phosphorylated form of H2AX is denoted as γH2AX). γH2AX then becomes the centre of a focus to which proteins such as p53 binding protein 1 (53BP1), mediator of mammalian DNA damage checkpoint 1 (MDC1) and BRCA1 are recruited. The presence of these proteins at the focus site can be detected after 1 minute. ATM and MRN are also incorporated into the focus but not until about 30 minutes after a cell is damaged, however they are still present at the site of the damage [50]. DNA-PKcs also causes phosphorylation of H2AX in a similar manner to that of its family member ATM [51].



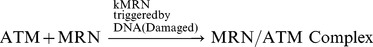


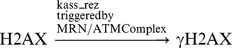


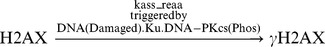


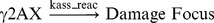


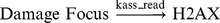


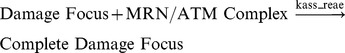


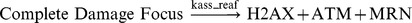



Following network construction the reaction rates were estimated using data from a variety of sources including our own experimentally determined rates of damage induction for the unstressed/not irradiated cells. See Table S2 and Table S3 for estimated molecule numbers, reaction rate constants and a comprehensive list of sources of experimental data. For a large number of individual reactions, kinetic rate constants were not available in the literature so we used available experimental time course data of recruitment and binding to calculate kinetic rate constants. For example, from [52] we know the average amount of Ku found in a eukaryotic cell (400000 molecules). We also know that after a DSB is formed Ku shows maximal recruitment at 3 minutes [53]. Combined with data of Ku rate of binding and dissociation to DNA [15], [31] we could estimate all the kinetic rates of Ku’s interaction with a DSB.

The model so far describes how a DNA double strand break at a single DNA site is formed and resolved. To create the final model we converted the single site model to SBML Shorthand [54] and edited it using a Python script to repeat the repair pathways and the flagging pathway multiple times to represent up to twenty sites of damage. This allowed us to simulate the creation and repair of multiple individual DSBs and their damage foci simultaneously. Twenty theoretical sites were chosen since in the live cell observations no more than fifteen breaks appeared at any one time. The SMBL Code of the model can be found in Model S1.

### Model Simulation and Analysis

The model was simulated using the Gillespie algorithm implemented within the stochastic simulator Gillespie2 [54], [55] in an unstressed state (not irradiated) and a stressed state (irradiated) 100 times each for 30 hours with 1 minute time points. The stressed state model was represented by increasing the rate of ROS production 2.5 times compared to the unstressed model, in line with observations of the relative amount of ROS in basal and stressed cells (The species ‘Source of Damage in the model which had a fixed constant value and is used in the reaction that produces ROS was increased 2.5 fold) [11]. After the initial simulations were carried out the dissociation reaction of Ku was modified to represent the observed change in Ku dissociation from a break site when in an oxidising environment [15].

We used an R script to extract the data from the individual simulation files and to calculate the longevity of individual damage foci whilst adjusting the output to account for transient foci by filling in time between a focus resolving and reforming if the duration was 20 minutes or less, in the same way as was done during the analysis of the live cell data.

To compare the live cell and *in silico* data sets we constructed histograms and Kaplan-Meier curves and carried out Cox Regression analysis (Type I error rate, alpha = 0.05).

### Ku 80 pKa Shift Analysis

Cysteine residues that are ionised at physiological pH have an increased susceptibility to oxidation and redox regulation [25]. To determine whether any of the cysteine residues within Ku 80 had this characteristic we carried out pK_a_ shift calculations. Two PDB files of the Ku70/80 heterodimer, one bound to DNA (PDB ID: IJEY) and the other free (PDB ID: IJEQ ) [39] were obtained from the RCSB Protein Data Bank (www.pdb.org) [56]. The X-ray crystal structures within the files were protonated and had atomic partial charges assigned using PDB2PQR [57], [58]. The structures were then used to calculate the free energy change of ionisation of the cysteine residues 157, 235, 249, 296, 346 and 493 in the protein environment and isolated in solution, using the Adaptive Poisson Boltzmann Solver (APBS) [59]. The obtained energy changes were then used to calculate each residue’s pK_a_ shift using the method described [60] and detailed on the APBS website www.poissonboltzman.org.

## Supporting Information

Table S1 Table of Reactions. The model considers a maximum of 20 damage foci and the index i identifies species associated with processes relating to individual foci; the addition of _2 is added to accommodate modification of species, e.g. DNA-PK sDSB Complexi_2 represents phosphorylated DNA-PK sDSB Complex.”
(PDF)

Table S2 Calculated initial molecule number for repair factors in the model with references.
(PDF)

Table S3 Table of kinetic rate constants used in model with references.
(PDF)

Model S1 SBML code for the full model.
(XML)

Video S1 Damage foci formation and resolution in unstressed MRC5 fibroblasts
(AVI)

Video S2 Damage foci formation and resolution in stressed MRC5 fibroblasts.
(AVI)
