# Supplementary material for: Systems Modelling of NHEJ Reveals the Importance of Redox Regulation of Ku70/80 in the Dynamics of DNA Damage Foci
Source: PLoS One. 2013 Feb 6;8(2):e55190. doi: 10.1371/journal.pone.0055190 (PMC3566652; doi:10.1371/journal.pone.0055190)
Supplement: Table S1 — Table of Reactions. The model considers a maximum of 20 damage foci and the index i identifies species associated with processes relating to individual foci; the addition of _2 is added to accommodate modification of species, e.g. DNA-PK sDSB Complexi_2 represents phosphorylated DNA-PK sDSB Complex.” (PDF) [file pone.0055190.s001.pdf]

Table S1. Table of Reactions. The model considers a maximum of 20 damage foci and the index i identifies species associated with processes relating to individual foci; the addition of _2 is added to accommodate modification of species, e.g. DNA-PK sDSB Complexi_2 represents phosphorylated DNA-PK sDSB Complex.

Reaction ID	Reaction Formulae
rea	Background Sources of Damage -> ROS
reb*i*	ROS + DNA*i* -> "Simple DSB*i*"
rec	ROS -> "ROS Degradation"
red*i*	uKu70/80 -> Ku70/80*i*; "Simple DSB*i*"
ree*i*	Ku70/80*i* -> uKu70/80
ref*i*	Simple DSB*i* + Ku70/80*i* -> "Ku70/80 sDSB Complex*i*"
reg*i*	Ku70/80 DSB Complex*i* -> "Simple DSB*i*" + Ku70/80*i*
reh*i*	uDNA-PKcs -> "DNA-PKcs *i*"; "Simple DSB*i*"
rei*i*	DNA-PKcs *i* -> "uDNA-PKcs "
rej*i*	Ku70/80 sDSB Complex*i* + "DNA-PKcs *i*" -> "DNA-PK sDSB Complex*i*"
reDNAPKa*i*	DNA-PK sDSB Complex*i* -> "Simple DSB*i*" + Ku70/80*i* + "DNA-PKcs *i*"
rek*i*	DNA-PK sDSB Complex*i* -> "DNA-PK sDSB Complex*i*_2"
reDNAPKb*i*	DNA-PK sDSB Complex*i*_2 -> "Simple DSB*i*" + Ku70/80*i* + "DNA-PKcs *i*"
rel*i*	uLiIV XRCC4 -> "LiIV XRCC4*i*"; "DNA-PK sDSB Complex*i*_2"
rem*i*	LiIV XRCC4*i* -> "uLiIV XRCC4"
ren*i*	DNA-PK sDSB Complex*i*_2 + "LiIV XRCC4*i*" -> "DNA-PK LiIV XRCC4 sDSB Complex*i*"
reo*i*	DNA-PK LiIV XRCC4 sDSB Complex*i* -> DNA*i* + Ku70/80*i* + "DNA-PKcs *i*" + "LiIV XRCC4*i*" + "sDSB Acurate Repair"
rep*i*	ROS + DNA*i* -> "Complex DSB*i*"
req*i*	uKu70/80 -> Ku70/80*i*; "Complex DSB*i*"
rer*i*	Complex DSB*i* + Ku70/80*i* -> "Ku70/80 cDSB Complex*i*"
res*i*	Ku70/80 cDSB Complex*i* -> "Complex DSB*i*" + Ku70/80*i*
ret*i*	uDNA-PKcs -> "DNA-PKcs *i*"; "Complex DSB*i*"
reu*i*	Ku70/80 cDSB Complex*i* + "DNA-PKcs *i*" -> "DNA-PK cDSB Complex*i*"
reDNAPKc*i*	DNA-PK cDSB Complex*i* -> "Complex DSB*i*" + Ku70/80*i* + "DNA-PKcs *i*"
rev*i*	DNA-PK cDSB Complex*i* -> "DNA-PK cDSB Complex*i*_2"
reDNAPKd*i*	DNA-PK cDSB Complex*i*_2 -> "Complex DSB*i*" + Ku70/80*i* + "DNA-PKcs *i*"
rew*i*	uLiIV XRCC4 -> "LiIV XRCC4*i*"; "DNA-PK cDSB Complex*i*_2"
rex*i*	DNA-PK cDSB Complex*i*_2 + "LiIV XRCC4*i*" -> "DNA-PK LiIV XRCC4 cDSB Complex*i*"
rey*i*	DNA-PK LiIV XRCC4 cDSB Complex*i* -> DNA*i* + Ku70/80*i* + "DNA-PKcs *i*" + "LiIV XRCC4*i*" + "cDSB Acurate Repair"
reMRNa*i*	ATM*i* + MRN*i* -> "MRN ATM Complex*i*"; "Simple DSB*i*"
reMRNb*i*	ATM*i* + MRN*i* -> "MRN ATM Complex*i*"; "Ku70/80 sDSB Complex*i*"
reMRNc*i*	ATM*i* + MRN*i* -> "MRN ATM Complex*i*"; "Complex DSB*i*"
reMRNd*i*	ATM*i* + MRN*i* -> "MRN ATM Complex*i*"; "Ku70/80 cDSB Complex*i*"
rez*i*	H2AX*i* -> H2AX*i*_2; "MRN ATM Complex*i*"
reaa*i*	H2AX*i* -> H2AX*i*_2; "DNA-PK sDSB Complex*i*_2"
reab*i*	H2AX*i* -> H2AX*i*_2; "DNA-PK cDSB Complex*i*_2"
reac*i*	H2AX*i*_2 -> "Damage Focus*i*"
read*i*	Damage Focus*i* -> H2AX*i*
reae*i*	Damage Focus*i* + "MRN ATM Complex*i*" -> "Complete Damage Focus*i*"
reaf*i*	Complete Damage Focus*i* -> H2AX*i* + ATM*i* + MRN*i*

reaq*i*	uPARP -> PARP*i*; "Simple DSB*i*"
rear*i*	PARP*i* -> uPARP
reas*i*	Simple DSB*i* + PARP*i* -> "sDSB PARP Complex*i*"
reat*i*	sDSB PARP Complex*i* -> "Simple DSB*i*" + PARP*i*
reau*i*	uLiIII -> LiIII*i*; "sDSB PARP Complex*i*"
reav*i*	LiIII*i* -> uLiIII
reaw*i*	sDSB PARP Complex*i* + LiIII*i* -> "sDSB PARP-LiIII Complex*i*"
reax*i*	sDSB PARP-LiIII Complex*i* -> DNA*i* + PARP*i* + LiIII*i* + "sDSB Acurate Repair (PARP)"
reay*i*	sDSB PARP-LiIII Complex*i* -> DNA*i* + PARP*i* + LiIII*i* + "Deleterious sDSB Repair"
reMRNg*i*	ATM*i* + MRN*i* -> "MRN ATM Complex*i*"; "sDSB PARP Complex*i*"
reaz*i*	uPARP -> PARP*i*; "Complex DSB*i*"
reba*i*	Complex DSB*i* + PARP*i* -> "cDSB PARP Complex*i*"
rebb*i*	cDSB PARP Complex*i* -> "Complex DSB*i*" + PARP*i*
rebc*i*	uLiIII -> LiIII*i*; "cDSB PARP Complex*i*"
rebd*i*	cDSB PARP Complex*i* + LiIII*i* -> "cDSB PARP-LiIII Complex*i*"
rebe*i*	cDSB PARP-LiIII Complex*i* -> DNA*i* + PARP*i* + LiIII*i* + "cDSB Acurate Repair (PARP)"
rebf*i*	cDSB PARP-LiIII Complex*i* -> DNA*i* + PARP*i* + LiIII*i* + "Deleterious cDSB Repair"
reMRNh*i*	ATM*i* + MRN*i* -> "MRN ATM Complex*i*"; "cDSB PARP Complex*i*"
reMRNi*i*	ATM*i* + MRN*i* -> "MRN ATM Complex*i*"; "DNA-PK DSB Complex*i*"
reMRNj*i*	ATM*i* + MRN*i* -> "MRN ATM Complex*i*"; "DNA-PK DSB Complex*i*_2"
reMRNk*i*	ATM*i* + MRN*i* -> "MRN ATM Complex*i*"; "DNA-PK LiIV XRCC4 DSB Complex*i*"
reMRNl*i*	ATM*i* + MRN*i* -> "MRN ATM Complex*i*"; "DNA-PK cDSB Complex*i*"
reMRNm*i*	ATM*i* + MRN*i* -> "MRN ATM Complex*i*"; "DNA-PK cDSB Complex*i*_2"
reMRNn*i*	ATM*i* + MRN*i* -> "MRN ATM Complex*i*"; "DNA-PK LiIV XRCC4 cDSB Complex*i*"
reMRNo*i*	ATM*i* + MRN*i* -> "MRN ATM Complex*i*"; "sDSB PARP-LiIII Complex*i*"
reMRNp*i*	ATM*i* + MRN*i* -> "MRN ATM Complex*i*"; "cDSB PARP-LiIII Complex*i*"
