# Supplementary material for: Systems Modelling of NHEJ Reveals the Importance of Redox Regulation of Ku70/80 in the Dynamics of DNA Damage Foci
Source: PLoS One. 2013 Feb 6;8(2):e55190. doi: 10.1371/journal.pone.0055190 (PMC3566652; doi:10.1371/journal.pone.0055190)
Supplement: Table S2 — Calculated initial molecule number for repair factors in the model with references. (PDF) [file pone.0055190.s006.pdf]

Table S2. Calculated initial molecule number for repair factors in the model with references.

Species	Starting Quantity (No. of Molecules)	Reference
Ku70/80	400000	[1]
DNA-PKcs	100000	[2]
LiIV-XRCC4	30000	[3,4]
PARP-1	1200000	[5-7]

1. Anderson CW, Carter TH (1996) The DNA-activated protein kinase -- DNA-PK. Curr Top Microbiol Immunol 217: 91-111.
2. Anderson CW, Lees-Miller SP (1992) The nuclear serine/threonine protein kinase DNA-PK. Critical reviews in eukaryotic gene expression 2: 283.
3. Leppard JB, Dong Z, Mackey ZB, Tomkinson AE (2003) Physical and Functional Interaction between DNA Ligase III and Poly(ADP-Ribose) Polymerase 1 in DNA Single-Strand Break Repair. Molecular and Cellular Biology 23: 5919-5927.
4. Windhofer F, Wu W, Iliakis G (2007) Low levels of DNA ligases III and IV sufficient for effective NHEJ. Journal of Cellular Physiology 213: 475-483.
5. D'Amours D, Desnoyers S, D'Silva I, Poirier GG (1999) Poly(ADP-ribosyl)ation reactions in the regulation of nuclear functions. Biochem J 342: 249-268.
6. Ludwig A, Behnke B, Holtlund J, Hilz H (1988) Immunoquantitation and size determination of intrinsic poly(ADP-ribose) polymerase from acid precipitates. An analysis of the in vivo status in mammalian species and in lower eukaryotes. Journal of Biological Chemistry 263: 6993-6999.
7. Yamanaka H, Penning CA, Willis EH, Wasson DB, Carson DA (1988) Characterization of human poly(ADP-ribose) polymerase with autoantibodies. Journal of Biological Chemistry 263: 3879-3883.
