# Supplementary material for: Systems Modelling of NHEJ Reveals the Importance of Redox Regulation of Ku70/80 in the Dynamics of DNA Damage Foci
Source: PLoS One. 2013 Feb 6;8(2):e55190. doi: 10.1371/journal.pone.0055190 (PMC3566652; doi:10.1371/journal.pone.0055190)
Supplement: Table S3 — Table of kinetic rate constants used in model with references. (PDF) [file pone.0055190.s002.pdf]

Table S3. Table of kinetic rate constants used in model with references.

Rate Constant	Value	Reaction	Source
kass_rea	0.001	Production of ROS	Experimental Data
kass_reb	0.15	Induction of a Simple DSB	Experimental Data, [1]
kass_rec	0.03	Degredation of ROS	Experimental Data
kass_red	0.6	Recruitment of Ku70/80 to sDSB	[2]
kass_ree	0.02	Ku70/80 leaving site of Damage	Model constant
kass_ref	5e-006	Ku70/80 binding to sDSB	[3]
kass_reg	0.5	Ku70/80 dissociation from sDSB	[3]
kass_reh	0.6	Recruitment of DNA-PKcs to sDSB	[4]
kass_rei	0.02	DNA-PKcs leaving site of Damage	Model constant
kass_rej	9e-005	DNA-PKcs binding to Ku70/80-sDSB Complex	[4]
kass_rek	0.3	DNA-PK Autophosphorylation	[4]
kass_rel	0.001	Recruitment of LiIV/XRCC4 to sDSB	[5]
kass_rem	0.2	LiIV/XRCC4 leaving site of Damage	Model constant
kass_ren	0.00035	LiIV/XRCC4 binding to DNA-PK-sDSB Complex	[5]
kass_reo	0.075	Ligation of sDSB and dismantling of Repair Complex	[5]
kass_rep	0.15	Induction of a Complex DSB	Experimental Data, [1]
kass_req	0.6	Recruitment of Ku70/80 to cDSB	[2,6]
kass_rer	4e-006	Ku70/80 binding to cDSB	[3,6]
kass_res	0.4	Ku70/80 dissociation from cDSB	[3]
kass_ret	0.6	Recruitment of DNA-PKcs to cDSB	[4,6]
kass_reu	7e-005	DNA-PKcs binding to Ku70/80-cDSB Complex	[4,6]
kass_rev	0.3	DNA-PK Autophosphorylation	[4,6]
kass_rew	0.001	Recruitment of LiIV/XRCC4 to cDSB	[5]
kass_rex	0.0002	LiIV/XRCC4 binding to DNA-PK-cDSB Complex	[5,6]
kass_rey	0.075	Ligation of cDSB and dismantling of Repair Complex	[5,6]
kMRN	0.75	Activation and Binding of ATM and MRN	[7,8]
kass_rez	0.5	H2AX Phosphorylation	[9]
kass_reaa	0.5	H2AX Phosphorylation	[9]
kass_reab	0.5	H2AX Phosphorylation	[9]
kass_reac	0.5	Damage Focus Formation	[10]
kass_read	0.1	Damage Focus Dismantling	[10]
kass_reae	0.5	ATM-MRN Complex binding to Damage Focus	[11]
kass_reaf	0.03	ATM-MRN-Damage Focus Dismantling	[11]
kass_reaq	0.6	Recruitment of PARP to sDSB	[3]
kass_rear	0.02	PARP leaving site of Damage	Model constant
kass_reas	5e-007	PARP binding to sDSB	[3]
kass_reat	0.02	PARP dissociation from sDSB	[3]
kass_reau	0.005	Recruitment of LiIII/XRCC3 to sDSB	[12,13]
kass_reav	0.02	LiIII/XRCC3 leaving site of Damage	Model constant
kass_reaw	0.00035	LiIII/XRCC3 binding to DNA-PK-sDSB Complex	[12,13]
kass_reax	0.0006	Accurate ligation of sDSB and dismantling of Repair Complex	[1,14]
kass_reay	0.0009	Inaccurate ligation of sDSB and dismantling of Repair Complex	[1,14]
kass_reaz	0.6	Recruitment of PARP to cDSB	[3]
kass_reba	4e-007	PARP binding to cDSB	[3]
kass_rebb	0.02	PARP leaving site of Damage	[3]

kass_rebc	0.005	Recruitment of LiIII/XRCC3 to cDSB	[12,13]
kass_rebd	0.0002	LiIII/XRCC3 binding to DNA-PK-cDSB Complex	[12,13]
kass_rebe	0.0006	Accurate ligation of cDSB and dismantling of Repair Complex	[1,14]
kass_rebf	0.0009	Inaccurate ligation of cDSB and dismantling of Repair Complex	[1,14]
kdiss_DNAPK	0.02	DNA-PK dissociation from DSB	[3,4]

1. Covo S, de Villartay J-P, Jeggo PA, Livneh Z (2009) Translesion DNA synthesis-assisted non-homologous end-joining of complex double-strand breaks prevents loss of DNA sequences in mammalian cells. Nucl Acids Res 37: 6737-6745.
2. Mari P-O, Florea BI, Persengiev SP, Verkaik NS, Brüggenwirth HT, et al. (2006) Dynamic assembly of end-joining complexes requires interaction between Ku70/80 and XRCC4. Proceedings of the National Academy of Sciences 103: 18597-18602.
3. Wang M, Wu W, Wu W, Rosidi B, Zhang L, et al. (2006) PARP-1 and Ku compete for repair of DNA double strand breaks by distinct NHEJ pathways. Nucl Acids Res 34: 6170-6182.
4. Uematsu N, Weterings E, Yano K-i, Morotomi-Yano K, Jakob B, et al. (2007) Autophosphorylation of DNA-PKCS regulates its dynamics at DNA double-strand breaks. J Cell Biol 177: 219-229.
5. Yano K, Chen DJ (2008) Live cell imaging of XLF and XRCC4 reveals a novel view of protein assembly in the non-homologous end-joining pathway. Cell cycle (Georgetown, Tex) 7: 1321.
6. Dobbs TA, Palmer P, Maniou Z, Lomax ME, O'Neill P (2008) Interplay of two major repair pathways in the processing of complex double-strand DNA breaks. DNA Repair 7: 1372-1383.
7. Celeste A, Fernandez-Capetillo O, Kruhlak MJ, Pilch DR, Staudt DW, et al. (2003) Histone H2AX phosphorylation is dispensable for the initial recognition of DNA breaks. Nat Cell Biol 5: 675-679.
8. Lukas C, Melander F, Stucki M, Falck J, Bekker-Jensen S, et al. (2004) Mdc1 couples DNA double-strand break recognition by Nbs1 with its H2AX-dependent chromatin retention. EMBO J 23: 2674-2683.
9. Leatherbarrow EL, Harper JV, Cucinotta FA, O'Neill P (2006) Induction and quantification of γ-H2AX foci following low and high LET-irradiation. International Journal of Radiation Biology 82: 111 - 118.
10. Schultz LB, Chehab NH, Malikzay A, Halazonetis TD (2000) P53 Binding Protein 1 (53bp1) Is an Early Participant in the Cellular Response to DNA Double-Strand Breaks. The Journal of Cell Biology 151: 1381-1390.
11. Cann KL, Hicks GG (2007) Regulation of the cellular DNA double-strand break response. Biochemistry and Cell Biology 85: 663-674.
12. Wang H, Rosidi B, Perrault R, Wang M, Zhang L, et al. (2005) DNA Ligase III as a Candidate Component of Backup Pathways of Nonhomologous End Joining. Cancer Research 65: 4020-4030.
13. Windhofer F, Wu W, Iliakis G (2007) Low levels of DNA ligases III and IV sufficient for effective NHEJ. Journal of Cellular Physiology 213: 475-483.
14. Ahmed EA, de Boer P, Philippens MEP, Kal HB, de Rooij DG (2009) Parp1-XRCC1 and the repair of DNA double strand breaks in mouse round spermatids. Mutation Research/Fundamental and Molecular Mechanisms of Mutagenesis 683: 84-90.
